# Mutation-induced dimerization of transforming growth factor-β–induced protein may drive protein aggregation in granular corneal dystrophy

**DOI:** 10.1016/j.jbc.2021.100858

**Published:** 2021-06-04

**Authors:** Nadia Sukusu Nielsen, Trine A.F. Gadeberg, Ebbe Toftgaard Poulsen, Seandean Lykke Harwood, Christian E. Weberskov, Jan Skov Pedersen, Gregers R. Andersen, Jan J. Enghild

**Affiliations:** 1 Department of Molecular Biology and Genetics, Science Park, Aarhus University, Aarhus, Denmark; 2 Department of Chemistry and Interdisciplinary Nanoscience Center (iNANO), Aarhus University, Aarhus, Denmark

**Keywords:** *TGFBI*, TGFBIp, granular corneal dystrophy, lattice corneal dystrophy, X-ray crystallography, protein cross-linking, protein aggregation, extracellular matrix protein, CD, corneal dystrophy, DSSO, disuccinimidyl sulfoxide, ECM, extracellular matrix, FAS1, fasciclin 1, GCD, granular corneal dystrophy, LCD, lattice corneal dystrophy, SAXS, small-angle X-ray scattering, SEC, size exclusion chromatography, *TGFBI*, transforming growth factor-β-induced gene, TGFBIp, transforming growth factor-β-induced protein

## Abstract

Protein aggregation in the outermost layers of the cornea, which can lead to cloudy vision and in severe cases blindness, is linked to mutations in the extracellular matrix protein transforming growth factor-β–induced protein (TGFBIp). Among the most frequent pathogenic mutations are R124H and R555W, both associated with granular corneal dystrophy (GCD) characterized by the early-onset formation of amorphous aggregates. The molecular mechanisms of protein aggregation in GCD are largely unknown. In this study, we determined the crystal structures of R124H, R555W, and the lattice corneal dystrophy-associated A546T. Although there were no changes in the monomeric TGFBIp structure of any mutant that would explain their propensity to aggregate, R124H and R555W demonstrated a new dimer interface in the crystal packing, which is not present in wildtype TGFBIp or A546T. This interface, as seen in both the R124H and R555W structures, involves residue 124 of the first TGFBIp molecule and 555 in the second. The interface is not permitted by the Arg124 and Arg555 residues of wildtype TGFBIp and may play a central role in the aggregation exhibited by R124H and R555W *in vivo*. Using cross-linking mass spectrometry and in-line size exclusion chromatography–small-angle X-ray scattering, we characterized a dimer formed by wildtype and mutant TGFBIps in solution. Dimerization in solution also involves interactions between the N- and C-terminal domains of two TGFBIp molecules but was not identical to the crystal packing dimerization. TGFBIp-targeted interventions that disrupt the R124H/R555W crystal packing dimer interface might offer new therapeutic opportunities to treat patients with GCD.

To date, 74 mutations in the transforming growth factor-β–induced (*TGFBI*) gene have been associated with the group of rare autosomal dominant diseases, *TGFBI*-linked corneal dystrophies (CDs) (1). The prevalence of *TGFBI*-linked CDs in the Chinese and Korean populations is 1 in 416 and 1 in 870, respectively (2, 3). The manifestation of this group of inherited disorders is blurred or cloudy vision and, in severe cases, blindness, caused by protein deposition in the cornea. The current treatments are temporary and involve invasive procedures such as corneal transplantation or laser surgery to remove the deposits. The *TGFBI*-linked CDs can be divided into different types depending on the phenotypic appearance of the deposits, localization, and age of onset. Lattice corneal dystrophy (LCD) is characterized by the appearance of amyloid plaques. In contrast, granular corneal dystrophy (GCD) comprises two subtypes, type 1 (GCD1) and type 2 (GCD2, Avellino), which are both associated with early-onset formation of amorphous protein aggregates. Additional lattice-like amyloid deposits appear in some patients with GCD2 (4). The principal constituent of the protein deposits is one of the most abundant proteins in the extracellular matrix (ECM) of the human cornea, transforming growth factor-β–induced protein (TGFBIp) (5, 6, 7, 8, 9, 10, 11, 12). The function of corneal TGFBIp is elusive, but its ability to bind both integrins and ECM molecules indicates a role in cell adhesion (1).

Many of the pathogenic TGFBIp mutations affect its structural stability (13, 14, 15). TGFBIp consists of an N-terminal CROPT domain followed by four fasciclin 1 (FAS1) domains (FAS1-1, FAS1-2, FAS1-3, and FAS1-4). The X-ray crystal structure of wildtype TGFBIp reveals an elongated banana-shaped molecule with the five domains arranged like pearls on a curved string. The CROPT and FAS1-1 domains are each linked to the FAS1-2 domain by an interdomain disulfide bond. The CROPT domain is organized around a central three-stranded β-sheet and a small β ribbon and has two intradomain disulfide bonds. The four FAS1 domains contain both α-helices and β-sheets arranged in the order α1-α3 + β1 + α4-α6 + β2-β8 (16). The 74 identified pathogenic mutations are distributed throughout the domains of TGFBIp, but not evenly; 57 are found within the FAS1-4 domain and ten within the FAS1-1 domain (1). Arg124 of the FAS1-1 domain and Arg555 of the FAS1-4 domain have been classified as mutational hot spots owing to the frequency of individual pathogenic mutations observed in patients at these sites. It is unclear how mutations at opposite ends of TGFBIp cause similar phenotypic outcomes, as seen for both LCD and GCD, although hypotheses involving several molecular pathways have been proposed (1, 5). In addition, several studies have investigated the multimerization of TGFBIp. A small-angle X-ray scattering (SAXS) study found that TGFBIp is a monomer at low concentrations but is prone to form dimers and trimers at high concentrations (11 μM). More dilute TGFBIp purified from human and porcine corneas was shown to be monomeric when analyzed by calibrated size exclusion chromatography (SEC) (17, 18). However, the oligomeric state of TGFBIp within the cornea is unknown, as are the potential effects of pathogenic mutations on TGFBIp multimerization.

It has been suggested that mutations associated with LCD lead to a structural destabilization of TGFBIp. This is reflected by an altered proteolytic turnover of TGFBIp in LCD (19), possibly involving the serine protease HtrA1, which promotes the formation of amyloidogenic peptides and thus amyloid deposits in the cornea (20). On the other hand, GCD-associated mutations decrease TGFBIp’s solubility and affect its interactions with corneal stroma components (1, 15, 20). In addition, TGFBIp in GCD deposits is less proteolytically processed than in LCD (5, 7).

In this study, we used limited proteolysis to evaluate the stability of wildtype TGFBIp and eight mutants. GCD-associated mutants were cleaved similarly to wildtype TGFBIp, whereas all but one of the LCD-associated mutants were more susceptible to proteolysis. Furthermore, we determined the crystal structures of the three TGFBIp variants R124H (GCD2), R555W (GCD1), and A546T (LCD). None of the three mutations had significant effects on either the overall conformation of TGFBIp or fold of the FAS1-1 and FA1-4 domains. However, residues 540 to 575 have higher average B-factors in the crystal structure of A546T, suggesting increased flexibility of these residues. In addition, a crystal packing interface present in the structures of both R124H and R555W that involves residue 124 in one molecule and 555 in another molecule may represent an intermolecular interaction that contributes to the aggregation of these mutants in GCD. This crystal packing interface explains why the R124H and R555W mutations, positioned at opposite ends of TGFBIp, lead to similar early-onset formation of amorphous aggregates of TGFBIp. Altogether, the present study shows that altered proteolytic processing and intermolecular interactions are likely to underlie the pathogenic mechanisms of LCD- and GCD-associated *TGFBI* mutations, respectively, rather than any major structural rearrangement of TGFBIp.

## Results

### LCD mutants are more susceptible to proteolysis than wildtype TGFBIp and GCD mutants

**Figure 1:**
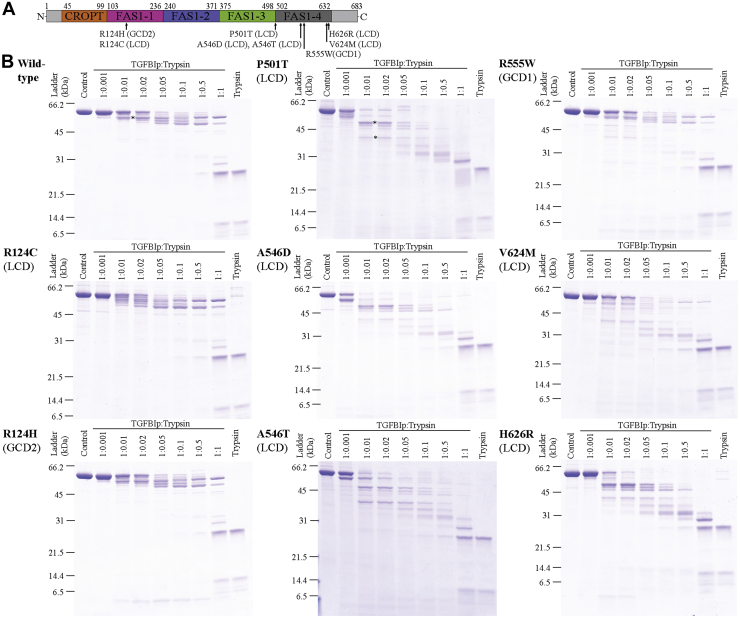
**LCD mutations induce changes in the proteolytic stability of TGFBIp.***A*, domain organization of TGFBIp indicating the location of the mutated residues. *B*, limited proteolysis of wildtype TGFBIp and eight mutants; 0.8 μM of TGFBIp wildtype and mutants was incubated with a titration series of trypsin for 90 min at 37 °C and subsequently analyzed by reducing SDS-PAGE. TGFBIp to trypsin ratios (w/w) are depicted at the top of the gels. The *asterisks* (∗) mark cleavage products with an intact N terminus as determined by Edman degradation. All the mutants associated with LCD, except R124C, were more readily degraded by trypsin than wildtype TGFBIp, whereas R555W and R124H associated with GCD were as stable as wildtype TGFBIp.

We recombinantly expressed and purified wildtype TGFBIp (residues 45–632) and eight mutants without the N- and C-terminal regions for crystallization screening. The eight TGFBIp mutants included R124C and R124H located in the FAS1-1 domain of TGFBIp, P501T found in the linker region between the FAS1-3 and FAS1-4 domain, and A546D, A546T, R555W, V624M, and H626R situated in the FAS1-4 domain (Fig. 1*A*). Two of the mutants, R124H and R555W, are associated with GCD2 and GCD1, respectively, whereas the rest of the mutants are linked to different LCD types (21). Prior studies revealed differences in the proteolytic stability of TGFBIp mutants depending on their CD phenotype, where LCD mutants (except R124C) were less stable than wildtype TGFBIp and GCD mutants were as stable as or more stable than wildtype TGFBIp (13, 14, 20). To determine whether the truncated TGFBIp constructs used in this study showed the same correlation, wildtype TGFBIp and mutants were subjected to a titration series of two proteases with nonoverlapping substrate preferences, trypsin (Fig. 1*B*) and chymotrypsin (Fig. S1), and the extent of cleavage was evaluated by reducing SDS-PAGE. The mutants associated with GCD were as resistant to proteolysis as wildtype TGFBIp. On the other hand, five of the six LCD-linked mutants were more susceptible to proteolysis than wildtype TGFBIp, most likely due to a destabilization of the FAS1-4 domain, as demonstrated previously (13, 14, 20). The only exception is the LCD-associated mutant R124C, which seemed as resistant as wildtype TGFBIp to proteolysis. Arg124 is surface exposed in the wildtype structure and located in the compact N-terminal half of TGFBIp (16). Edman degradation of the major initial cleavage products of wildtype and P501T TGFBIp demonstrated an intact N terminus (*i.e.*, with the sequence: GPNVCAVQKVI), suggesting they are initially cleaved at sites located toward the C terminus of TGFBIp (Fig. 1*B*). The localization of Arg124 in the three-dimensional structure as well as trypsin’s preferential cleavage in the C terminus of TGFBIp might explain why the R124C mutation does not seem to change the proteolytic stability of TGFBIp.

### Crystal structures of GCD and LCD mutants

To investigate whether structural changes might explain the aggregation of TGFBIp mutants, we pursued structure determination of the eight TGFBIp mutants by X-ray crystallography. However, only the structures of the TGFBIp mutants R124H, A546T, and R555W could be determined (Table 1). The structures of R124H and R555W were determined at a resolution of 2.7 Å and 2.0 Å, respectively, and therefore accurately revealed details in changes around the mutated residues. As a lower resolution of 4.8 Å was obtained for the A546T mutant, structural differences between this mutant and wildtype TGFBIp may not be significant.

**Table 1:** Data collection and refinement statistics prepared with phenix.table_one

Structure	R124H	A546T	R555W
PDB entry code	7AS7	7ASC	7ASF
Resolution range	45.85–2.65 (2.745–2.65)	60.94–4.801 (4.973–4.801)	49.24–2.0 (2.071–2.0)
Space group	C 2 2 21	P 41 21 2	C 2 2 21
Unit cell	77.37 93.87 214.02 90 90 90	206.76 206.76 127.54 90 90 90	78.06 98.49 207.84 90 90 90
Unique reflections	23,038 (2262)	14,000 (1376)	54,442 (5356)
Multiplicity	6.9 (7.1)	13.0 (14.1)	10.4 (10.5)
Completeness (%)	99.77 (99.60)	99.69 (100.00)	99.82 (99.61)
Mean I/σ(I)	7.71 (0.88)	11.10 (0.83)	8.38 (0.79)
R-merge	0.2056 (2.021)	0.1385 (2.901)	0.1883 (2.57)
CC_1/2_	0.996 (0.365)	0.999 (0.388)	0.998 (0.531)
Reflections used in refinement	23,008 (2254)	13,979 (1376)	54,362 (5338)
Reflections used for R-free	1998 (196)	1400 (138)	1998 (197)
R-work	0.2402 (0.4111)	0.2546 (0.3737)	0.2059 (0.4583)
R-free	0.2867 (0.4333)	0.2685 (0.3721)	0.2470 (0.4790)
Protein residues	584	1176	592
RMS bonds (Å)	0.009	0.007	0.016
RMS angles (°)	1.26	1.41	1.47
Ramachandran favored, allowed, outliers (%)	95.5	95.7	96.6
	3.6	3.5	3.4
	0.9	0.8	0.0
Clash score	2.56	2.09	2.08

Statistics for the highest-resolution shell are shown in parentheses.

**Figure 2:**
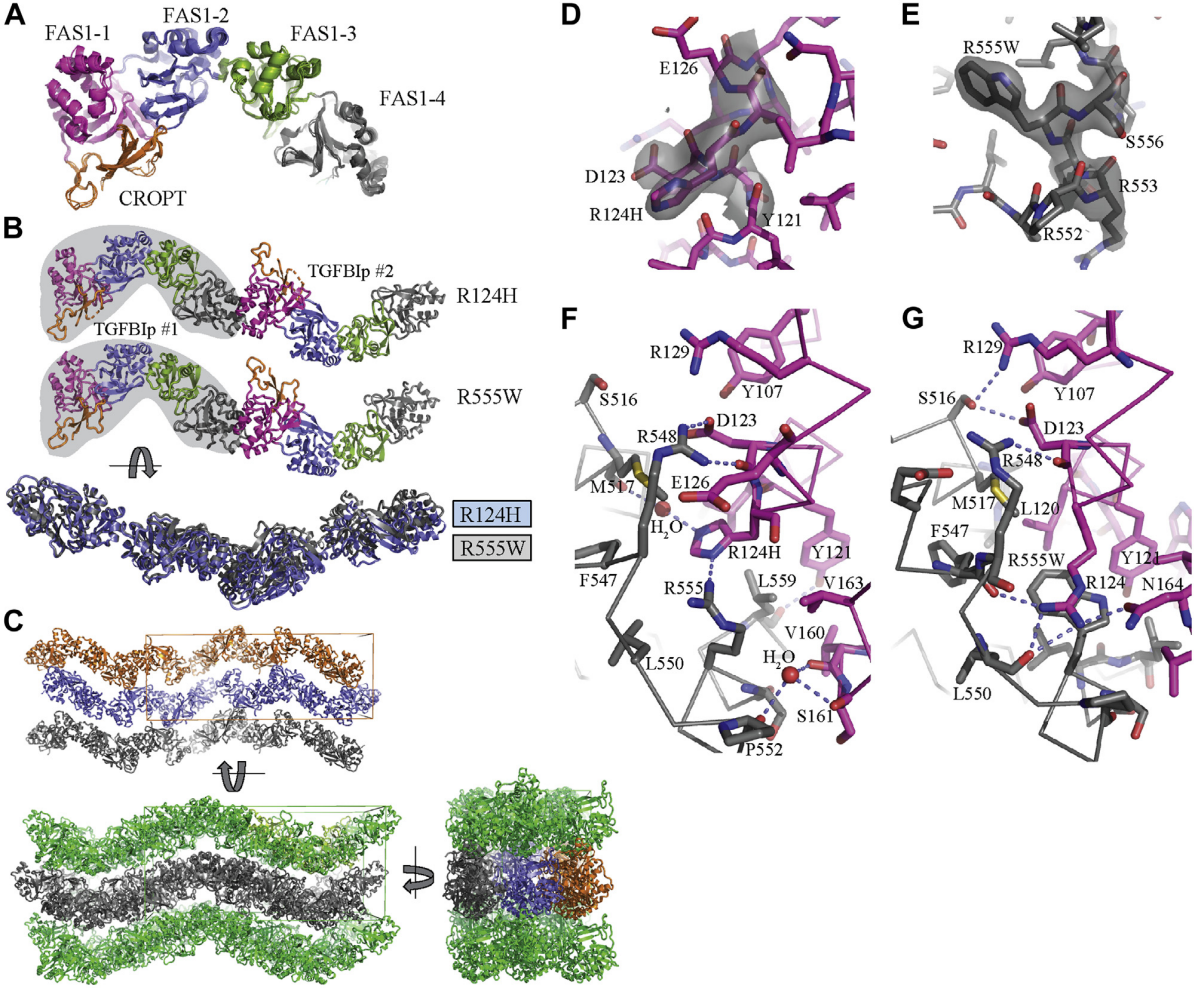
**Structures of TGFBIp R124H and R555W reveal an intermolecular interaction involving the mutated residues.***A*, comparison of the structures of the three TGFBIp mutants with chain B in the structure of wildtype TGFBIp (Protein Data Bank entry 5NV6). *B*, the highly similar FAS1-1:FAS1-4 dimers present in the crystal lattices formed by TGFBIp R124H and R555W. *C*, in the crystal lattice, infinite linear chains of the dimers in *B* align in antiparallel layers both horizontally and vertically, *D*, omit 2mF_o_-DF_c_ electron density for the R124H structure contoured at 1.4 σ. Residues 122 to 126 were omitted for map calculation. *E*, omit 2mF_o_-DF_c_ electron density for the R555W structure contoured at 1.4 σ. Residues 553 to 557 were omitted for map calculation. *F*, details of the intermolecular interface in the crystal packing FAS1-1:FAS1-4 dimer observed for TGFBIp R124H. Carbon atoms in FAS1-1 (TGFBIp #1) and FAS1-4 (TGFBIp #2) are colored *magenta* and *gray*, respectively. *Dotted lines* indicate hydrogen bonds and electrostatic interactions formed across the interface. *G*, details of the intermolecular interface for the R555W structure presented as in *F*. Notice the absence of water molecules and the stacking interaction between the R124 side chain from one molecule and the R555W side chain from the second molecule.

The unit cell parameters of R124H and the R555W are highly related, reflecting very similar crystal packing in these structures with one TGFBIp molecule in the asymmetric unit. In contrast, the asymmetric unit of the A546T structure contains two molecules, and the unit cell parameters are not related to those of the two other mutants or the unit cell for the wildtype structure. The overall conformations of the three TGFBIp mutants are close to one of the two conformations present in the crystal structure of wildtype TGFBIp (16), differing by a hinge-like rotation of 12° of the FAS1-3 and FAS1-4 domains. The root-mean-square deviations between the three mutant structures and this wildtype conformation (chain B in Protein Data Bank [PDB] entry 5NV6) is 1.0 to 1.6 Å for 577 C_α_ atoms (Fig. 2*A*). In summary, none of the three mutations appears to have significant, crystal packing-independent effects on either the overall conformation of TGFBIp or the fold of the FAS1-1 and FAS1-4 domains harboring the three studied mutations.

Mutations of Arg124 in FAS1-1 and Arg555 in FAS1-4 are the most frequent pathogenic mutations (22). A striking feature found in both the R124H and R555W structures and involving their mutated residues is a crystal packing interface between the FAS1-1 and FAS1-4 domains of different TGFBIp molecules, where residue 124 interacts with residue 555 in a second TGFBIp molecule (*i.e.*, His124 and Arg555 in R124H, Arg124 and Trp555 in R555W). The dimensions of the resulting dimer are 226 × 73 × 62 Å as compared with average dimensions of 125 × 68 × 41 Å for the monomer structures, due to the head-to-tail arrangement of the banana-shaped TGFBIp monomers (Fig. 2*B*). In the crystal packing, an infinite arrangement of R124H or R555W monomers resulted in linear chains of monomers running parallel to a crystallographic 2_1_ axis, in which neighboring mutant TGFBIp molecules are related by a 180° rotation and a translation of 107 or 104 Å along the symmetry axis. Such linear monomer chains form antiparallel layers in both the horizontal and the vertical direction (Fig. 2*C*).

Interface analysis with PISA (23) showed that the buried surface in the intermolecular FAS1-1:FAS1-4 interface described above is 574 Å^2^ in R124H and 808 Å^2^ in R555W and is not present in either the A546T or the wildtype structures. The FAS1-1:FAS1-4 interface in the R555W structure is significantly more hydrophobic than in the R124H structure, with a Δ^i^G *p*-value of 0.11 and 0.40, respectively. This R555W FAS1-1:FAS1-4 interface is also by far the most hydrophobic in all the four TGFBIp crystal structures determined to date and has a calculated Δ^i^G of −9 kcal/mol, which is also the most favorable value of all the intermolecular contacts present in these structures. Of note, the three structures of R124H, A546T, and R555W share another larger (>1500 Å^2^) crystal packing interface, but this interface is quite polar, is split into two patches, and does not involve residue 124 or 555. Furthermore, the largest intermolecular interfaces in the original wildtype structure are not present in our structures of mutant TGFBIp. A comparison of all available structures reveals that the R124H mutation has no influence on the structure for the FAS1-1 domain (Fig. S2*A*), whereas in both the R124H and the R555W structures, the α-helix harboring residue 555 is shifted relative to the position of the helix in the wildtype structure (Fig. S2*B*). Since both wildtype and R124H structures have an arginine residue at position 555, the shift of the α-helix is not due to the R555W mutation. Instead, the position of the α-helix is likely to be determined by the intermolecular contact with the FAS1-1 domain. For both R124H and R555W, the electron density around the mutated side chain is well defined (Fig. 2, *D* and *E*). This allows an unambiguous modeling of how the mutated side chain interacts with its surroundings, including the neighboring monomer and water molecules. In the R124H structure, the FAS1-1 His124 is in direct contact with Arg555 through a side-chain hydrogen bond (Fig. 2*F*). His124 also engages in a second water-bridged interaction with the main chain of Met517. The side chain of Tyr121 also forms a hydrogen bond with the main chain of Leu559 in the second TGFBIp molecule. In addition, multiple hydrophobic interactions involving side chains of Tyr121, Leu120, Met517, Phe547, Leu550, and Leu559 surround His124 and Arg555. In the much more hydrophobic R555W interface, there are no water molecules close to Trp555 (Fig. 2*G*). The interface is organized around the Trp555 side chain, and similarly to the R124H interface, the tryptophan indole ring is surrounded by a hydrophobic shell of side chains contributed by Phe547, Leu550, Leu558, Leu559, Leu120, Y121, Val163, and Leu167. A crucial interaction sealing the pocket around Trp555 is made by Arg124 from the second TGFBIp molecule, as its guanidinium group stacks with the tryptophan indole ring in a cation–π interaction. In addition, the Arg124 side chain further stabilizes the intermolecular interaction through hydrogen bonds with the main-chain carbonyl groups of Leu550 and Phe547. The Arg124 and Arg555 residues in wildtype TGFBIp do not permit this intermolecular interface due to size and electrostatic repulsion between the two arginines. This explains why this interface is not observed in the crystal structures of A546T and wildtype TGFBIp. In summary, our structures of TGFBIp R124H and R555W suggested an intermolecular dimerization that requires mutations in either of the two residues, which appeared to be especially stable for R555W. No alternative explanation is suggested by our structures, and we therefore propose that this intermolecular contact triggers the aggregation of TGFBIp observed in individuals possessing these two mutations.

**Figure 3:**
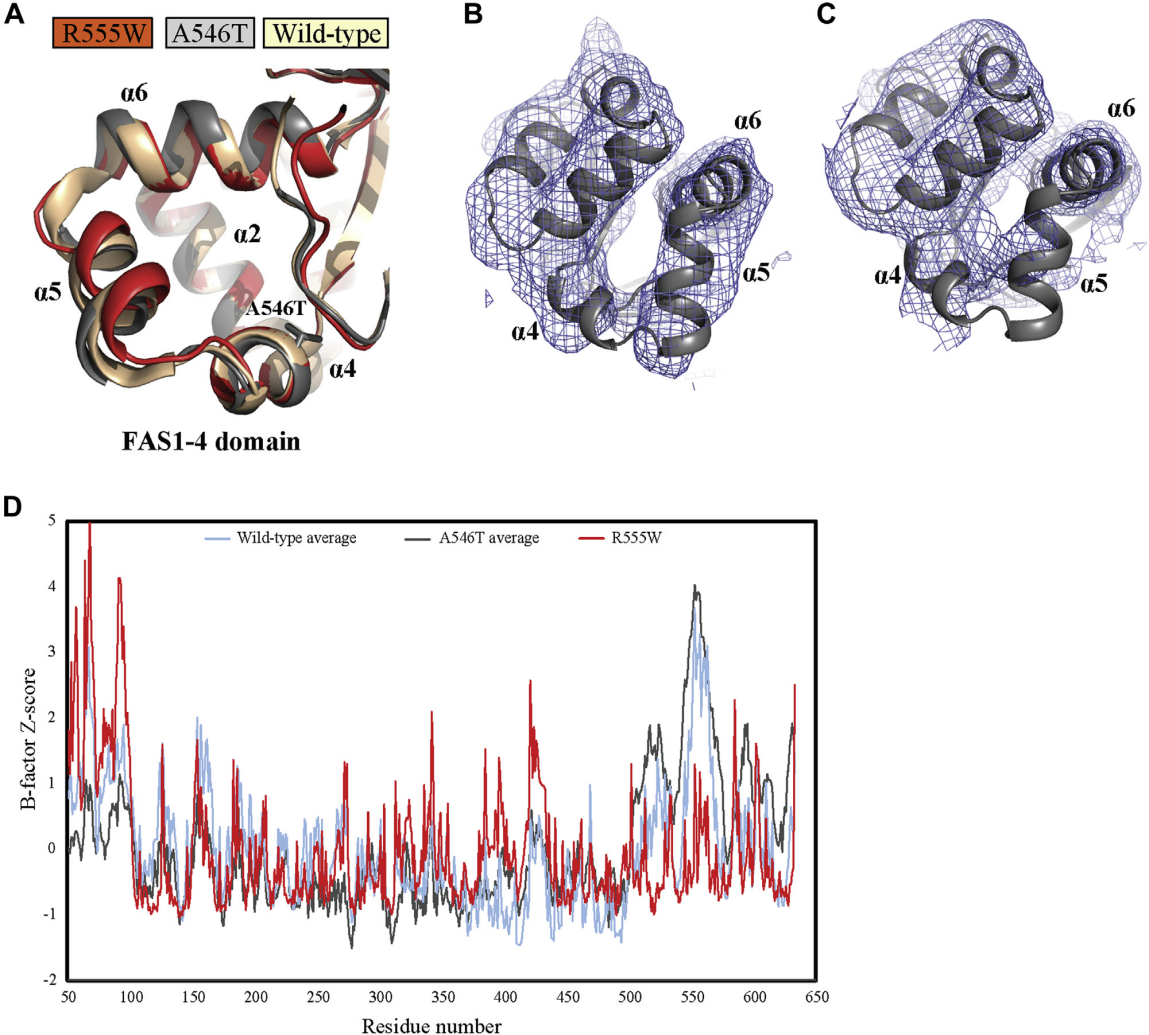
**The structure of TGFBIp A546T supports increased mobility within the FAS1-4 domain.***A*, a comparison of the FAS1-4 domains in three structures does not reveal significant structural changes induced by mutation of alanine 546 to threonine. *B* and *C*, the 2mF_o_-DF_c_ electron density contoured at 1σ around the A546T FAS1-4 domain of the two copies present in the asymmetric unit. The density is weak, especially the α5 helix located downstream of the mutated residue compared with other helices in the FAS1-4 domain. *D*, Z-score plot of residue B-factors for all selected structures of TGFBIp. In the A546T structure, B-factors in the FAS1-4 domain are generally higher than expected compared with other structures, suggesting this region is more flexible or structurally heterogeneous in the presence of the A546T mutation. The low B-factors in the R555W structure are likely due to its strong crystal interaction with a neighboring FAS1-1 domain.

Crystal packing contacts shared by R124H and R555W offer an explanation for their aggregation phenotype, whereas the enhanced susceptibility of A546T toward proteolytic degradation was not immediately explainable from its structure. The low resolution of the data prevents the identification of detailed structural changes induced by the additional methyl and hydroxyl groups of Thr546. Nevertheless, a comparison of FAS1-4 domains from the structures available suggests that the overall location of the α5 helix–containing residues Arg553–Gly560 is altered by the A546T mutation (Fig. 3*A*). The significance of this difference is difficult to evaluate as the electron density for the α5 helix is weak compared with other parts of the structure, especially in chain B of the A546T structure (Fig. 3, *B* and *C*). However, an analysis of residue B-factors (*i.e.*, temperature factors) in all available TGFBIp structures suggests that large regions in the FAS1-4 domain, particularly residues 540 to 575, have higher average B-factors than expected in the presence of the A546T mutation (Fig. 3*D*). Elevated B-factors in a region of a protein usually correlate with higher mobility or multiple conformations of this region. If the elevated B-factors reflect a higher mobility or conformational heterogeneity of residues 540 to 575 *in vivo*, this would explain the phenotype and the increased susceptibility to proteolytic degradation.

### Characterization of soluble TGFBIp dimers

**Figure 4:**
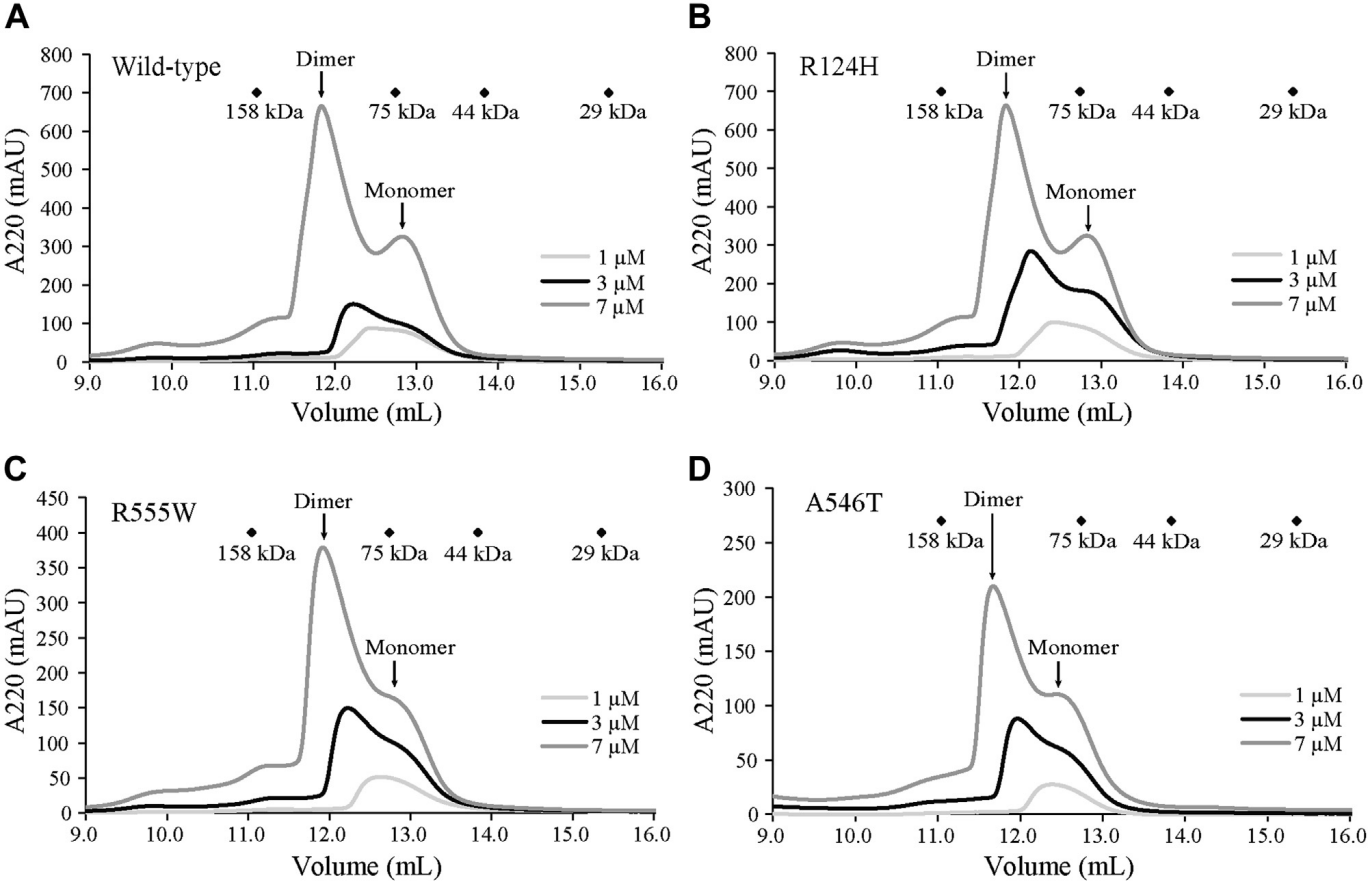
**TGFBIp dimerization in solution is concentration dependent.***A*, wildtype, *B*, R124H, *C*, R555W, and *D*, A546T TGFBIp at concentrations of 1, 3, or 7 μM were subjected to calibrated SEC. Size markers for calibration are indicated: 158 kDa (aldolase), 75 kDa (conalbumin), 44 kDa (ovalbumin), and 29 kDa (carbonic anhydrase). A concentration-dependent dimerization of TGFBIp is observed for both wildtype and mutants.

Recently, we determined the concentration of wildtype TGFBIp in the healthy cornea to be approximately 26 μM (1.7 mg/ml) (1). Roughly 60% of TGFBIp in the cornea is covalently bound to the ECM, and therefore, only a fraction of TGFBIp in the cornea is found in solution (24). To investigate whether TGFBIp dimers of R124H and R555W, as seen in the crystal packing, also exist in solution at concentrations comparable with the TGFBIp concentration *in vivo*, we analyzed the multimeric state of TGFBIp wildtype, R124H, A546T, and R555W by calibrated SEC on a Superdex 200 column (Fig. 4). At 7 μM, the majority of TGFBIp wildtype and mutants existed as dimers in solution (Fig. 4). The dimerization was concentration dependent, as the majority of TGFBIp wildtype and mutants were monomeric at 1 μM. The same concentration-dependent dimerization is observed for wildtype TGFBIp and all the mutants. This suggests that the dimers observed in our SEC experiments are different from the FAS1-1:FAS1-4 dimer formed in the crystal packing of R124H and R555W.

### The soluble TGFBIp dimer is arranged in a head-to-tail configuration

**Figure 5:**
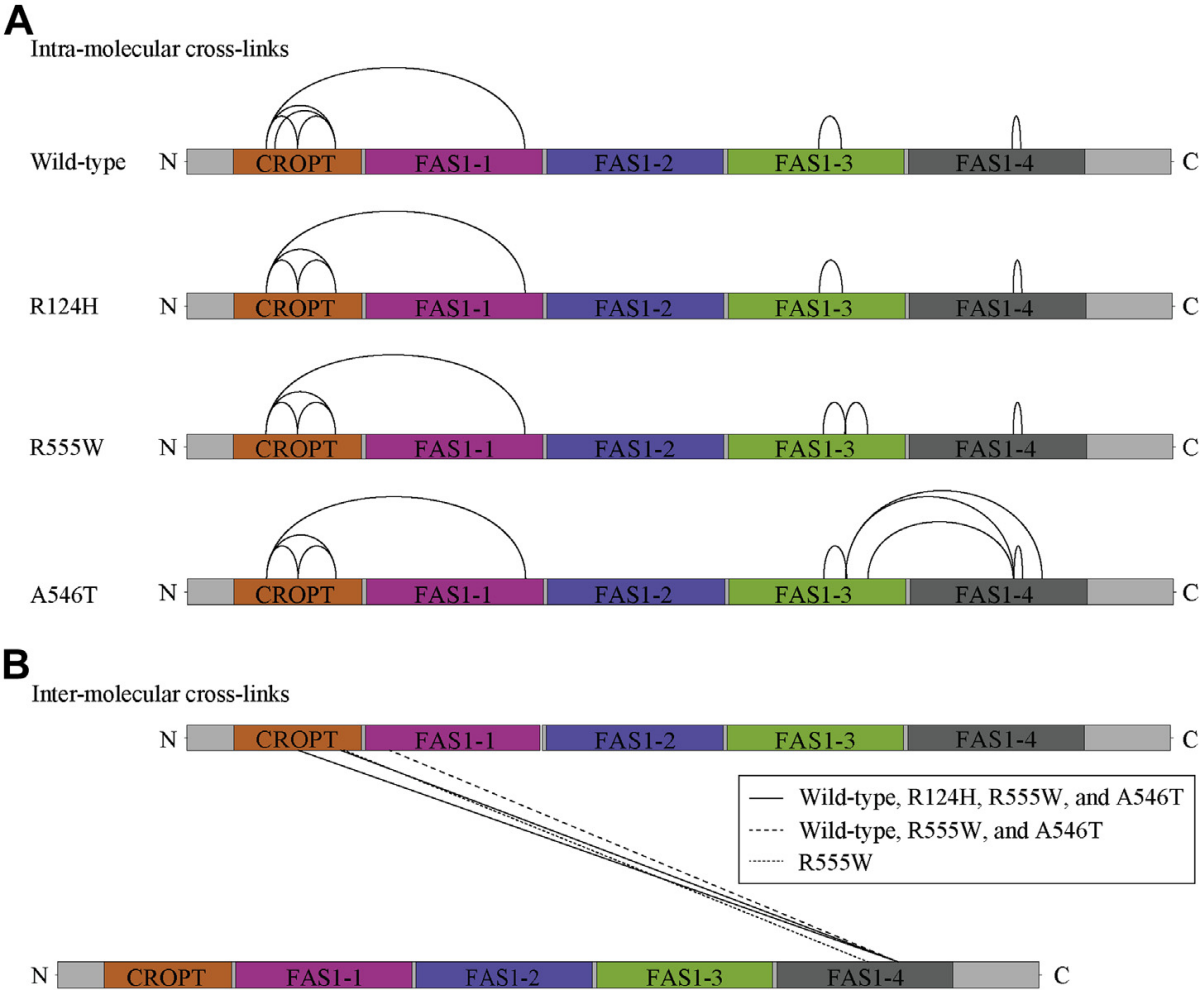
**Visualization of intra- and intermolecular cross-links identified for wildtype TGFBIp and mutants.** DSSO-cross-linked monomer and dimer bands of wildtype TGFBIp and mutants were digested into peptides and analyzed by LC-MS^3^. *A*, intramolecular cross-links and *B*, intermolecular cross-links identified in a minimum of two of three samples are depicted. Intermolecular cross-links involve the CROPT/FAS1-1 domain and the FAS1-4 domain, indicating an interaction between the N-terminal part of one molecule and the C-terminal part of another.

To identify the intermolecular interface within the soluble dimers, TGFBIp wildtype and mutants were cross-linked with disuccinimidyl sulfoxide (DSSO), an amine-reactive MS-cleavable cross-linker. DSSO primarily cross-links lysine residues with a maximum inter-α-carbon distance of 26 Å (25). DSSO-cross-linked samples were analyzed by reducing SDS-PAGE, and TGFBIp dimers, trimers, and higher-order multimers were observed (Fig. S3). Gel bands containing TGFBIp monomers and dimers were excised, digested with trypsin and GluC proteases, and analyzed by LC-MS^3^ to identify intra- and intermolecular cross-links (Fig. 5). Only cross-links identified in at least two of three replicate samples were included in further interpretation (Fig. 5*A* and Table S1). Seven intramolecular cross-links were identified for wildtype and R555W TGFBIp, six for R124H, and nine for A546T. Three cross-links connecting the FAS1-3 and FAS1-4 domains were exclusively identified for A546T. The higher flexibility of regions within the FAS1-4 domain that was suggested by the B-factor analysis explains the unique cross-linking of A546T in these regions.

Cross-links that were exclusively identified in the dimer band (in at least two of three samples) were classified as intermolecular cross-links (Fig. 5*B* and Table S2). Three intermolecular cross-links were identified for wildtype and A546T TGFBIp, two for R124H, and four for R555W. All of these cross-links indicate that the dimers arise from interactions between the N-terminal domains of one TGFBIp molecule (CROPT or FAS1-1) and the FAS1-4 domain of a second molecule. There are no clear differences between the cross-links formed between molecules in the soluble dimers of wildtype TGFBIp and mutants. Two intermolecular cross-links were identified for both R124H and R555W that link Lys72 with Lys596 and Lys90 with Lys596 (Table S2). The distances between the α-carbons of these cross-linked lysine residues in the crystal packing dimers of R124H and R555W were 54.1 Å (Lys72 to Lys596) and 45.4 Å (Lys90 to Lys596). These distances are not compatible with the maximum 26 Å length of the cross-linker, indicating that the soluble dimers are different from the crystal packing dimer. However, the dimerization of TGFBIp in solution through interactions between the N-terminal CROPT/FAS1-1 domains and the FAS1-4 domain could precede the crystal packing–type dimerization of R124H and R555W, which would then lead to aggregation and dystrophic corneal deposition *in vivo*.

### In-line SEC-SAXS confirms the presence of dimers in solution

In order to confirm the cross-linking mass spectrometry data in-line SEC-SAXS was performed on wildtype TGFBIp and both GCD mutants. The data processing is described in detail in Supplementary Methods, and UV and SAXS SEC profiles (Fig. S4), buffer-subtracted SAXS data (Fig. S5), Guinier plots (Fig. S6), scaled SAXS data (Fig. S7), distance–distance distribution functions (Fig. S8), and forward scattering and radius of gyration (Table S3) are found in the Supplementary Information.

Initial rigid-body modeling of the data sets with the most representative molecular size (radius of gyration of 46–47 Å) showed that the scattering for these data sets corresponds to that of a dimer. However, an end-to-end dimer, as present in the crystal packing of R124H and R555W, was too elongated to fit the SAXS data in all three cases.

**Figure 6:**
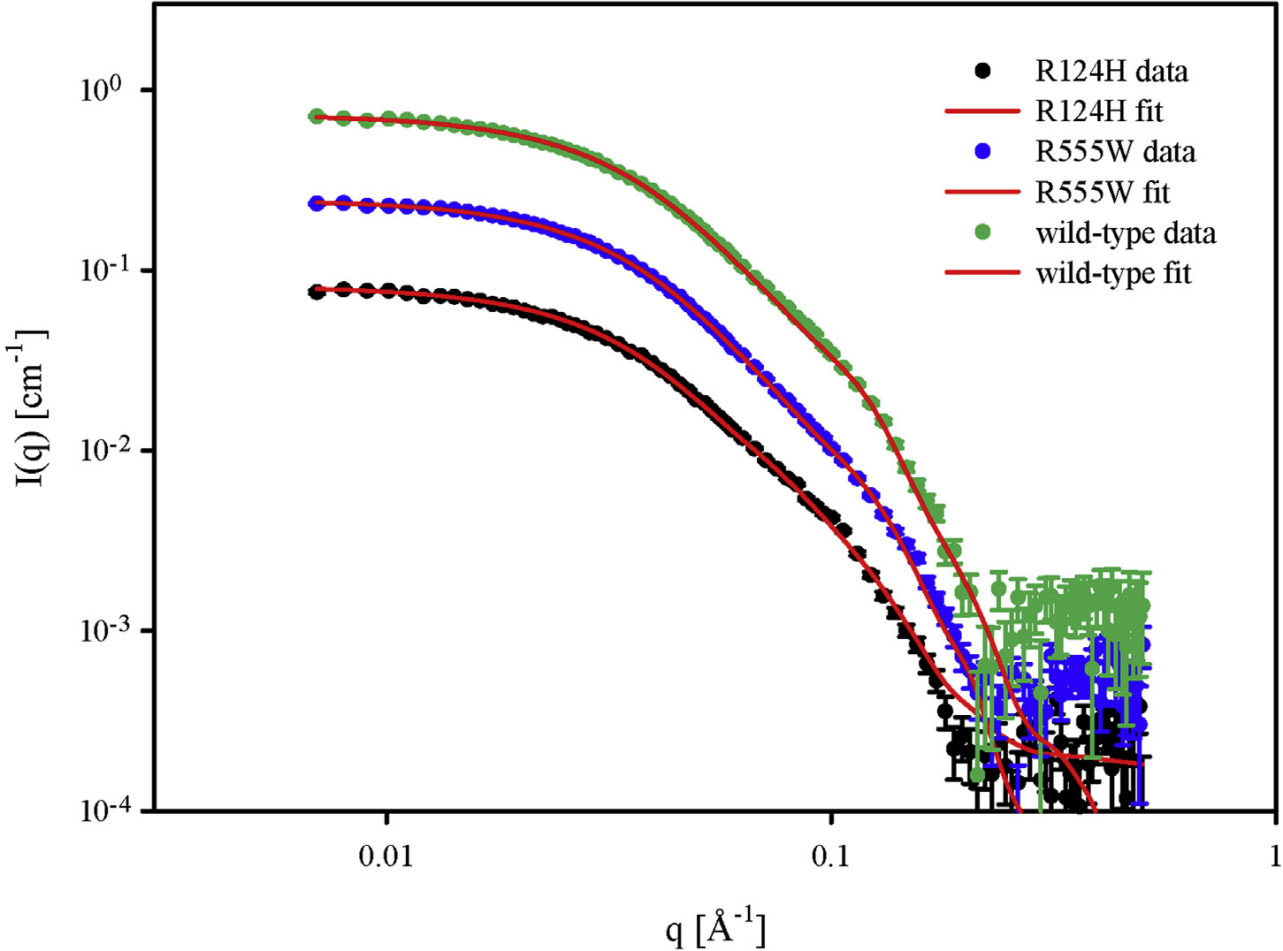
**SAXS data and fits for “N-in-arch closed coplanar/noncoplanar” structures for the most representative model.** Data and fits are normalized to 1.0 mg/ml. The data and fit for R555W are displaced upward by a factor of 3 and for wildtype by a factor of 9. The good fits to the data by the dimer models (Fig. 7) optimized by rigid-body refinement confirm that the solution structures are different from the elongated dimer structure in the crystal structure of R124H and R555W in agreement with XL-MS.

Instead, side-by-side dimers from the crystal structures packing were used in subsequent rigid-body modeling, using the identified intermolecular cross-links as restraints. For each data set, 50 runs were performed, and the output models were analyzed as clusters of similar models. In general, the clusters where the N terminus of one monomer is placed inside the arc of the other monomer, either coplanar or noncoplanar, have the best agreement with the SAXS data. The fit to the SAXS data for the most representative model in the clusters is shown in Figure 6. However, other clusters with other models fit the data nearly equally well suggesting that there is variation in the dimer structure in solution. The values of the restrained distances and the averaged reduced χ^2^ values of the largest clusters are displayed in Table S4. Of importance, none of these clusters of models has the end-to-end dimer interface found in the crystal structure of R124H and R555W.

**Figure 7:**
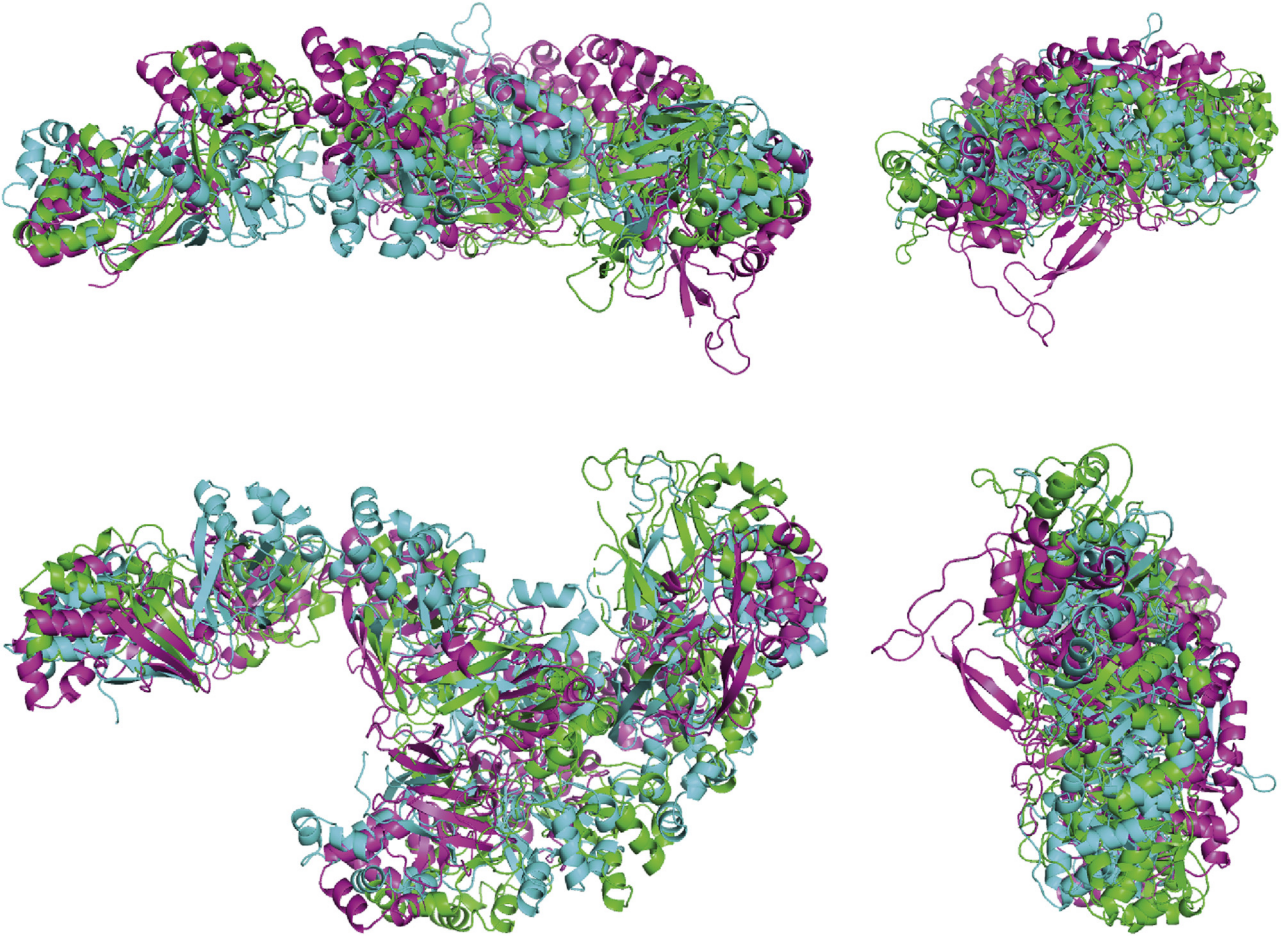
**Most representative structure for the clusters “N-in-arch closed coplanar/non-coplanar” for R124H (*green*), R555W (*light blue*) and wildtype (*purple*).** The optimized solution models are similar and more compact than the elongated dimer structure in the crystal structure of R124H and R555W.

The most representative models for R124H, R555W, and wildtype have been aligned and are displayed in Figure 7. Considering the low resolution of SAXS, these are highly similar models.

The SAXS analysis demonstrates that the dimer structure is more stable than other oligomeric structures in the concentration range of ∼0.5 to 1 mg/ml at which SAXS measurements took place. This predominance of TGFBIp dimers suggests that in-solution dimerization is poorly compatible with further polymerization; the interactions leading to in-solution dimers may prevent additional intermolecular interactions, perhaps through steric hindrance or conformational change.

## Discussion

### Mutations lead to phenotype-specific changes in the proteolytic stability of TGFBIp

TGFBIp mutants representing different phenotypes associated with *TGFBI*-linked CDs were expressed, purified, and subjected to limited proteolysis by the two complementary proteases, trypsin, and chymotrypsin. Trypsin degraded both wildtype TGFBIp and mutants; however, LCD mutants (except R124C) were more readily proteolyzed than wildtype and GCD mutants. Trypsin cleaves at the C-terminal side of basic residues, which are surface exposed in the structure of TGFBIp. However, trypsin is generally better able to access and cleave at dynamic or unstructured sites. On the other hand, chymotrypsin cleaves C-terminally to hydrophobic residues, which are buried in the structure of TGFBIp. Wildtype and GCD mutants were very resistant to proteolysis by chymotrypsin, whereas chymotrypsin readily degraded P501T, A546D, A546T, and V624M associated with LCD (Fig. S1). Hence, this further supports that LCD-associated mutations increase the dynamics of TGFBIp and expose its hydrophobic interior, compared with wildtype TGFBIp and GCD-associated mutants. If the increased susceptibility of most LCD-associated mutants to proteolysis *in vitro* is representative of a difference in their corneal processing *in vivo*, this would further indicate a crucial difference between the LCD and GCD aggregation pathways.

The results presented here do support other studies of the stability of TGFBIp mutants. In a study by Stenvang *et al.*, (15) LCD mutants displayed reduced thermal stabilities, whereas GCD mutants with surface-exposed mutated residues showed melting temperatures comparable with wildtype. Hence, neither changes in the thermal stability nor proteolytic processing can explain why GCD mutants are more prone to aggregation than wildtype TGFBIp.

### Residues 124 and 555 are potentially directly involved in the aggregation of R124H and R555W TGFBIp

The currently proposed molecular mechanisms of GCD involve decreased solubility of TGFBIp and/or altered interactions between TGFBIp and the components of the corneal stroma. However, no compelling results have been presented that explain why the two GCD mutations R124H and R555W at opposite and distal ends of the TGFBIp molecule lead to similar deposition in the cornea. This could be explained by the crystal packing dimers of R124H and R555W presented in this study. In the case of GCD2, His124 in one TGFBIp molecule interacts with Arg555 in another, whereas in the case of GCD1, Arg124 in one molecule interacts with Trp555 in another. In addition, the FAS1-1:FAS1-4 interface involves multiple hydrophobic interactions by surrounding residues. This interaction is repeated indefinitely in the crystal packing and generates linear monomer chains. This might resemble *in vivo* aggregation, as crystalline or discrete rod-shaped deposits have been observed with electron microscopy in corneas from patients with GCD (26, 27). A more detailed analysis of these deposits might reveal whether the FAS1-1:FAS1-4 dimerization interface occurs physiologically, although the heterogeneity from other corneal proteins will complicate such analyses and patient samples are rare.

### Future treatments of GCD

If the interactions present in the crystal packing for R124H and R555W indeed are important during their *in vivo* aggregation, this interface would be an important therapeutic target to prevent the aggregation of TGFBIp. Potential drugs could be small molecules or antibodies that bind the FAS1-1 or FAS1-4 patches and disrupt this intermolecular interaction. A compound interfering with the dimerization of both the R124H and R555W TGFBIp variants could be used to treat both patients with GCD1 and with GCD2.

The tendency of wildtype TGFBIp and mutants to form dimers in solution was investigated by calibrated SEC, cross-linking-MS, and in-line SEC-SAXS. From the cross-linking-MS and in-line SEC-SAXS results, it was evident that the dimers observed in the crystal packing of R124H and R555W are different from the dimers observed in solution. During *in vivo* aggregation of TGFBIp, the crystal packing dimer interface might become more favorable than the dimer interface observed in solution.

The calibrated SEC revealed a concentration-dependent dimerization where most of the TGFBIp in solution is monomeric at 1 μM, whereas at higher concentrations (7 μM) closer to those found *in vivo*, TGFBIp is mostly dimeric. A similar concentration dependence of the dimers of the crystal packing might exist. Partial reduction of the *in vivo* concentration of TGFBIp in the human cornea might then be a potential strategy for decreasing or delaying protein aggregation in patients with GCD. This could be carried out using siRNA or CRISPR-Cas9 to target the mutant allele(s) (28, 29). The fact that mice deficient in TGFBIp have a mostly normal phenotype (30) supports the possibility of reducing TGFBIp in the cornea without major side effects in terms of the structural integrity of the cornea. This is further supported by recent findings in transgenic TGFBI^R124H^ mice lacking the ability to form protein aggregates, most likely due to lower TGFBIp expression as seen for wildtype mice (31).

## Conclusion

This study contributes to the understanding of the molecular mechanisms involved in protein aggregation in GCD1 and GCD2, some of the most frequent *TGFBI*-linked corneal dystrophies. The crystal structures of R124H and R555W did not reveal any significant changes in the structure of the two TGFBIp variants that could explain their tendency to aggregate. However, from their crystal packing, we suggest that interactions involving residues 124 and 555 of two different TGFBIp molecules facilitate the aggregation of mutated TGFBIp. Future studies should be aimed at investigating whether this interaction occurs *in vivo*. If these interactions do in fact contribute to pathogenesis, drugs that target this dimer interface might be developed to prevent aggregation of TGFBIp.

## Experimental procedures

### Expression and purification of TGFBIp

A cDNA clone encoding N- and C-terminally truncated wildtype human TGFBIp (residues 45–632) with a C-terminal 6xHis tag was generated by GenScript from a previously described cDNA clone of full-length human TGFBIp (32). From this clone, the following mutants were prepared by site-directed mutagenesis by GenScript: R124C, R124H, P501T, A546D, A546T, R555W, V624M, and H626R. Expression was induced in the human cell line Freestyle 293-F (Invitrogen) using the polyethylenimine transfection method. Polyethylenimine and plasmid were mixed in a 3:1 ratio and preincubated at room temperature in expression medium (Gibco FreeStyle 293, Invitrogen) without penicillin/streptomycin for 20 min before being added to the cells. Prior to transfection, the cell density was adjusted to 1.0 × 10^6^ cells/ml in medium with penicillin/streptomycin. After 3 days, the medium was harvested for protein purification. Expression medium containing TGFBIp was dialyzed against two volumes of 12 l of buffer A (50 mM Tris-HCl, 100 mM NaCl pH 7.6) at 4 °C. The cell culture medium was centrifuged at 3000*g* for 10 min and supplemented with 20 mM imidazole before being applied to a 5-ml HisTrap HP column (GE Healthcare) equilibrated in 50 mM Tris-HCl, 100 mM NaCl, 20 mM imidazole pH 7.6. Protein was eluted with buffer B (50 mM Tris-HCl, 100 mM NaCl, 500 mM imidazole pH 7.6) and monitored at 280 nm along with manual collection of protein. The purity of the sample was assessed by SDS-PAGE, and the sample was subsequently dialyzed against PBS.

### Limited proteolysis

TGFBIp wildtype and mutants were subjected to a trypsin and chymotrypsin titration series. TGFBIp, 0.8 μM, was incubated with 0 to 2.1 μM of bovine pancreatic trypsin or 0 to 4 μM of bovine pancreatic chymotrypsin for 90 min at 37 °C in PBS, after which the proteases were inhibited by 2 mM PMSF for 15 min.

### SDS-PAGE

Samples were boiled for 5 min in SDS (1%) and 35 mM DTT. Denaturing SDS-PAGE was performed using a homemade 12% (w/v) or 5% to 15% (w/v) acrylamide gel and the discontinuous ammediol/glycine buffer system (33). Gels were stained with Coomassie Brilliant Blue.

### N-terminal sequencing of initial cleavage products

Samples of wildtype and P501T TGFBIp were incubated with trypsin as previously described, separated by reducing SDS-PAGE, and transferred to a polyvinylidene difluoride membrane. The major initial product bands were excised and applied to TFA-treated glass fiber membranes. Automated Edman degradation was performed on a PPSQ-31B protein sequencer (Shimadzu Biotech) with in-line phenylthiohydantoin analysis on an LC-20AT HPLC system. Data were obtained using Shimadzu PPSQ-31B software, and the sequences were identified manually from the UV 269 nm chromatograms.

### Crystallization of TGFBIp

R124H, R555W, and A546T TGFBIp samples were concentrated to ∼2 mg/ml using a Vivaspin two centrifugal concentrator unit with a 30-kDa cut-off (GE Healthcare). The concentrated protein was subjected to a final purification step by SEC on a Superdex 200 Increase 10/300 GL column (GE Healthcare) equilibrated with buffer C (20 mM Tris-HCl, 200 mM NaCl, pH 7.4). Fractions of 0.5 ml were collected and subsequently analyzed by SDS-PAGE. Pure and homogenous samples were pooled and concentrated to 3.5 to 5 mg/ml, followed by centrifugation for 5 min immediately prior to crystallization. The three TGFBIp mutants were screened for crystallization in sitting-drop vapor diffusion experiments using the commercial screens PEGRx, SaltRx, and PEGIon (Hampton Research). Drops of 150 nl TGFBIp mixed with 150 nl reservoir solution were formed with an Oryx4 (Douglas Instruments) crystallization robot and equilibrated against 50 μl reservoir solution in 96-well Swissci MRC crystallization plates at room temperature. Optimized reservoir conditions contained 0.1 M DL-Malic acid pH 7.0, 12% w/v PEG 3350 for R124H TGFBIp and 0.5 M Succinic acid pH 7.0, 0.1 M BIS-Tris propane pH 7.0 for R555W TGFBIp. One condition for A546T TGFBIp was further optimized using an additive screen (Hampton Research) and microseeding, and the final optimal reservoir condition contained 25 mM BIS-TRIS propane pH 9.0, 5% v/v PEG-mme 550, 1% PEG 3350.

### Structure determination

For data collection, crystals were soaked into the reservoir solution with added 25% to 30% (v/v) ethylene glycol as cryoprotectant followed by flash cooling in liquid nitrogen. Diffraction data were collected on the PETRA III beamlines P13 and P14 (EMBL Hamburg). The diffraction data were processed using XDS (34), and the resolution cut-off was determined according to the CC1/2 criterion (35). The structure of wildtype TGFBIp (PDB entry 5NV6) was used for molecular replacement in phenix.phaser (36). Models were manually built in Coot (37). Refinement was carried out in phenix.refine (38) with parameters rigid body, TLS, individual sites, and B-factors refinement. Data collection refinement statistics and Protein Data Bank entry codes are presented in Table 1.

### Cross-linking MS

Cross-linking of TGFBIp was performed in PBS, 6.7% v/v dimethyl sulfoxide using 3.1 μM TGFBIp and 155 μM DSSO (Thermo Fisher Scientific) in triplicate. Cross-linking was carried out for 30 min at room temperature and quenched by 25 mM Tris-HCl pH 8.8. After cross-linking, samples were reduced with 7 mM DTT for 15 min and alkylated with 21 mM iodoacetamide for 15 min at room temperature. Samples were run on 5% to 15% SDS-PAGE gels, and the relevant bands were excised. The bands were shrunk in acetonitrile, rehydrated in 50 mM ammonium bicarbonate pH 8, and digested 18 h with 1:30 (w/w) MS-grade porcine pancreatic trypsin (Thermo Fisher Scientific) at 37 °C. Additional digestion using 1:30 (w/w) endoproteinase GluC (New England Biolabs) was carried out for 4 h at 37 °C. Prior to MS analysis, peptides were desalted using Empore SPE Disks of C18 octadecyl packed in 10-μl pipet tips.

LC-MS^3^ peptide analysis was performed using on-line separation of peptides on a column pulled and packed in-house with Reprosil-Pur 120 C18-AQ 3 nM resin (Dr Maisch GmbH) on an EASY-nLC 1200 (Thermo Fisher Scientific) and an Orbitrap Eclipse Tribrid mass spectrometer (Thermo Fisher Scientific) with an MS^2^_MS^3^ method (39). Peptides were fragmented by 20% energy collision-induced dissociation at the MS^2^ level. If the unique mass difference of DSSO fragment doublets, Δm = 31.9715 Da, was observed, these fragments were isolated for MS^3^ using 35% energy collision-induced dissociation.

Cross-linked peptides were identified in the resulting raw files with Proteome Discoverer v. 2.4. (Thermo Fisher Scientific) and the add-on XLinkX node v. 2.4 using established workflows for the analysis of MS-cleavable cross-links (39). Databases containing wildtype or mutant TGFBIp sequences were searched. Precursor mass tolerance was set to 10 ppm, MS2 fragment mass tolerance was 20 ppm, and MS3 fragment mass tolerance was 0.5 Da. Cysteine carbamidomethylation was included as a fixed modification, and methionine oxidation was included as a variable modification. A maximum FDR of 1% and three missed cleavages for trypsin and GluC were allowed. Cross-links identified in at least two of the three replicates were depicted in the TGFBIp sequence using xiNET (40).

### Analytical SEC

Wildtype TGFBIp and mutants at a concentration of 1, 3, or 7 μM were subjected to calibrated SEC on a Superdex 200 Increase 10/300 GL column (GE Healthcare) equilibrated with PBS.

### In-line SEC-SAXS

Three samples of, respectively, R124H, R555W, and wildtype TGFBIp were measured at the BM29 BioSAXS beamline at the European Synchrotron Radiation Facility in Grenoble, France. The samples were concentrated just before being applied to an in-line Superdex Increase 200 3.2/300 SEC column equilibrated in 20 mM Tris, 150 mM NaCl, pH 7.4 (TBS), run at 0.1 ml/min. The absorbance at 280 nm was recorded using a Shimadzu UV spectrophotometer through a 1.0-mm path-length cell. The SAXS data sets were collected continuously with an acquisition time of 2.0 s and a delay time of 0.100 s between each frame.

The data were processed by standard beamline software (41) to obtain the azimuthally averaged intensity, *I*(*q*), *versus* scattering vector modulus, *q*. Further details on the processing are given in the Supplementary Methods in the Supplementary Information. The data were normalized using the scattering for bovine serum albumin at 1.0 mg/ml concentration to give an intensity scale in kilodaltons, when data are normalized by concentration in milligram per milliliter units. The SAXS elution profiles were constructed by integrating the first 100 points at low *q* in the azimuthally averaged non-background-subtracted data sets as this part of the data displayed the largest variation. The SAXS and UV elution profiles are displayed in Fig. S4. This and the following data processing and analysis were all done using home-written software.

SAXS frames from the region before the protein eluted were selected for representing buffer scattering, and frames from the protein peak were selected for representing five fractions for the sample scattering and buffer/background-subtract data were calculated (Fig. S5). In addition, the scattering from a large species that eluted late was subtracted from the sample scattering fractions. The data were rebinned to be approximately equidistant on a logarithmic *q* scale for improving the statistics at high *q*. Data quality of the final background-corrected data was checked by Guinier plots of ln(*I*(*q*)) *versus q*^2^ (Fig. S6). The data for each sample were scaled together at high *q*, where possible oligomerization has little influence on the data. This provides a correction for the concentration variation between the data and allows variation at low *q* due to possible oligomerization to be observed clearly (Fig. S7). Indirect Fourier transformation of the scaled data sets was performed (42, 43). This procedure yielded the pair distance distribution function *p*(*r*), which is a histogram over distances between pair of points within the particles. This function provides information on the maximum diameter of the particles (Fig. S8); the radius of gyration, *R*_*g*_; and the forward scattering, *I*(0) (Table S3). Inspection of the functions and values of the parameters allowed the identification of the fractions that contain the most stable oligomers for each sample, and the SAXS data sets for these fractions were used in further analysis.

Rigid-body modeling was performed using the in-house program described in (44, 45). Input models based on PDB entries 7AS7, 7ASG, and 5NV6 were used for the data for R124H, R555W, and wildtype, respectively. Optimizations without distance restraints were not able to distinguish the relative orientations of monomers as the monomer is quite symmetric within the low resolution of SAXS. Therefore, the identified cross-links were considered as restraints. It was decided only to include the two that occur in all three samples and in the A546T mutant, namely, the intermolecular Lys-Lys links 72 to 596 and 90 to 596, whereas the other possible intermolecular cross-links 127 to 596 and 90 to 563 distances were just calculated. The distance of the restraints was set to 31.0 Å to allow for dynamics and flexibility, and it was only distances larger than this that were included in the cost function with a parabolic dependence. It should be noted that examination of possible structures for a dimer suggest that cross-links 127 to 596 and 90 to 563 are hardly compatible with the cross-links 72 to 596 and 90 to 596, which could be due to variations in dimer structure, so that the cross-links do not all occur in the same dimer.

As the rigid-body refinement is performed with a random search, 50 runs were performed for each data set to check for variations in the structure. The resulting models were analyzed with respect to clusters of similar models using the program DAMCLUST from the ATSAS package (46) and during this procedure, the models in the clusters are aligned using DAMSUP also from the ATSAS package (47).

## Data availability

The mass spectrometry raw data and Proteome Discoverer result files have been deposited to the ProteomeXchange Consortium *via* the PRIDE (39) partner repository with the data set identifier PXD022976.

## Supporting information

This article contains supporting information.

## Conflict of interest

The authors declare that they have no conflicts of interest with the contents of this article.

## Supporting information

Supplementary Methods, Figures S1–S8 and Tables S1–S4

Supplementary Spreadsheet 1
