# Supplementary material for: Mutation-induced dimerization of transforming growth factor-β–induced protein may drive protein aggregation in granular corneal dystrophy
Source: J Biol Chem. 2021 Jun 4;297(1):100858. doi: 10.1016/j.jbc.2021.100858 (PMC8220419; doi:10.1016/j.jbc.2021.100858)
Supplement: Supplementary Methods, Figures S1–S8 and Tables S1–S4 [file mmc1.pdf]

Supplementary information

Mutation-induced dimerization of transforming growth factor-β-induced protein (TGFBIp) may drive protein aggregation in granular corneal dystrophy

Nadia Sukusu Nielsen[1], Trine A. F. Gadeberg[1], Ebbe Toftgaard Poulsen[1], Seandean Lykke Harwood[1], Christian E. Weberskov[1], Jan Skov Pedersen[2], Gregers R. Andersen[1], Jan J. Enghild[1*]

[1]Department of Molecular Biology and Genetics, Science Park, Aarhus University, Aarhus, Denmark
[2]Department of Chemistry and Interdisciplinary Nanoscience Center (iNANO), Aarhus University, Aarhus, Denmark

Supplementary methods……………………………………………………………………….2
Figure S1. **Limited proteolysis of wild-type TGFBIp and eight mutants**...................................... 4
Figure S2. **Mutation to R124H and R555W does not induce major structural changes**.............. 5
Figure S3. **SDS-PAGE of DSSO cross-linked TGFBIp wild-type and mutants**........................... 5
Figure S4. **Elution profiles from UV absorbance at 280 nm (black curve) and SAXS**…………..6
Figure S5. **Buffer-subtracted SAXS data**………………………………………………………...7
Figure S6. **Guinier plots of SAXS data**…………………………………………………………...8
Figure S7. **Plot of scaled SAXS data**………………………………………………………………9
Figure S8. **Distance distance distribution function *p*(*r*) for the scaled data**……………………...10
Table S1. **Intra-molecular cross-links**………………………………..11
Table S2. **Inter-molecular cross-links**………………………………..11
Table S3. **Values for forward scattering (arbitrary scale) and radius of gyration (Å)**…………..12
Table S4. **In-line SEC-SAXS parameters for most representative models**……………………12

Other supplementary information

Supplementary Spreadsheet 1 - Contains information on all cross-links identified by XL-MS.

MS data – The mass spectrometry raw data and Proteome Discoverer result files have been deposited to the ProteomeXchange Consortium via the PRIDE partner repository with the dataset identifier PXD022976.

3-D structures – The crystal structures have been deposited to the RCSB Protein Data Bank with the protein data bank entry codes 7AS7 (R124H), 7ASC (A546T), and 7ASF (R555W).

Supplementary methods

In-line SEC-SAXS experiment: Data processing and analysis

SAXS elution profiles were constructed by integrating the first 100 points at low q in the azimuthally averaged non-background-subtracted data sets, as this part of the data displayed the largest variation. The UV and SAXS profiles for the in-line SAXS experiments are shown on arbitrary scale in Fig. S4. For each of the samples, the profiles agree well, however, with some more smearing of the SAXS profiles probably due to smearing of the profiles in the system along the flow path. Five fractions were considered on the low-mass side of the main peak for frames 450-470, 470-490, 490-510, 510-530, and 530-550, and the SAXS data for these frames were averaged. For the buffer background scattering, 50-100 frames in the range of frames 100-200 were averaged, and were subtracted from the sample data fractions. The corresponding intensities are shown in Fig. S5. The data for the three samples look qualitatively similar with an expected relative intensity variation due to different protein concentrations for the different fractions. For all samples, a small upturn is observed at low q, which becomes more pronounced for the lower concentrations. This suggests pollution of the data by an aggregated species. Inspections of the chromatograms in Fig. S4 show that the level of both the UV and SAXS traces are higher after the main peak than before. This agrees that the aggregated species is interacting with the column and eluting late. In order to subtract the scattering from this, a buffer-subtracted data set for the large species was produced for each of the three samples. This data set was subtracted with a varying scale (in the range 0.5 – 1.0) in order to get a flat behavior at low q in log-log representation. The resulting data display linear behavior in a Guinier plot of $\ln(I(q))$ versus q^2 (Fig. S6). The scaled data are displayed in Fig. S7. They display variations at low q in agreement with a variation in oligomerization state. For the R124H sample, which overall had a relatively low concentration when applied to the SEC column, the data are nearly constant for the first three fractions and then decrease, whereas for R555W and wild-type, which both had an overall high concentration when applied to the SEC column, the data have large changes for the three first fractions and then become more constant.

The $p(r)$ functions from indirect Fourier transformation are shown in Fig. S8. The particles have a maximum diameter of about 160 Å and there is a general trend that the position of the maximum values shifts to lower distances with decreasing concentration in agreement with the particles becoming less globular and elongated. The R_g and $c\, I(0)$ values are displayed in table S3, where c is the protein concentration. The particles in the first three fractions of R124H and the last two fractions of R555W and wild-type have an R_g of about 47 Å, suggesting that there is a relatively stable species

in these fractions. This is further confirmed by the relatively small variation of $c\,I(0)$ in the first three fraction of R124H and the last two fractions of R555W and wild-type. The $p(r)$ function of the third fraction for R124H, the fourth fraction for R555W, and the fifth fraction for wild-type are shown in Fig. S8 and they show good agreement. The SAXS data set for these fractions were used for further modeling.

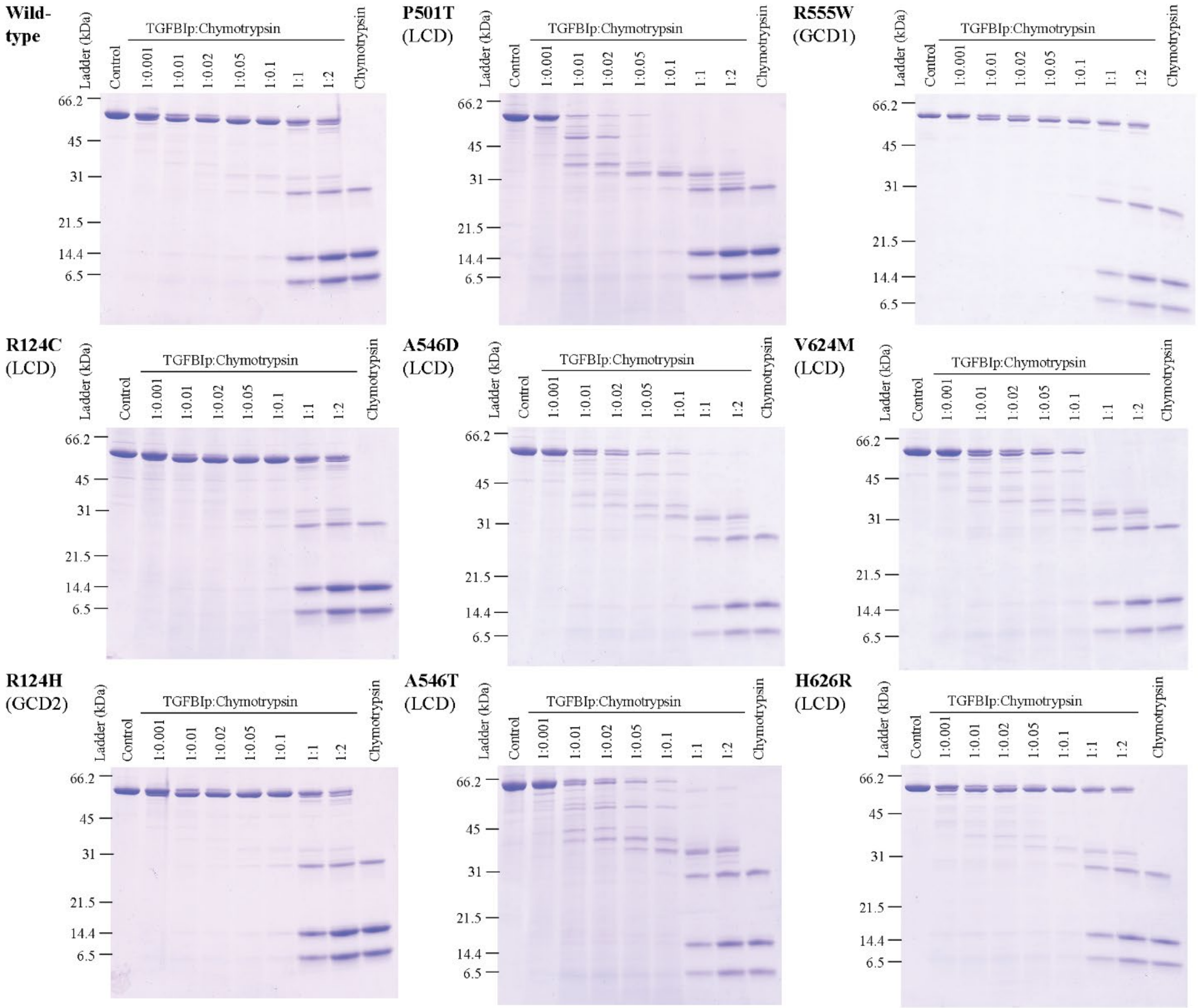

Figure S1. Limited proteolysis of wild-type TGFBIp and eight mutants. 0.8 µM of wild-type TGFBIp and mutants were incubated with a titration series of chymotrypsin for 90 min at 37 °C, and subsequently analyzed by reducing SDS-PAGE. TGFBIp to chymotrypsin ratios (w/w) are depicted at the top of the gels. All the mutants associated with LCD, except R124C, were more readily degraded by chymotrypsin compared to wild-type TGFBIp, whereas R555W and R124H associated with GCD were as resistant as wild-type TGFBIp.

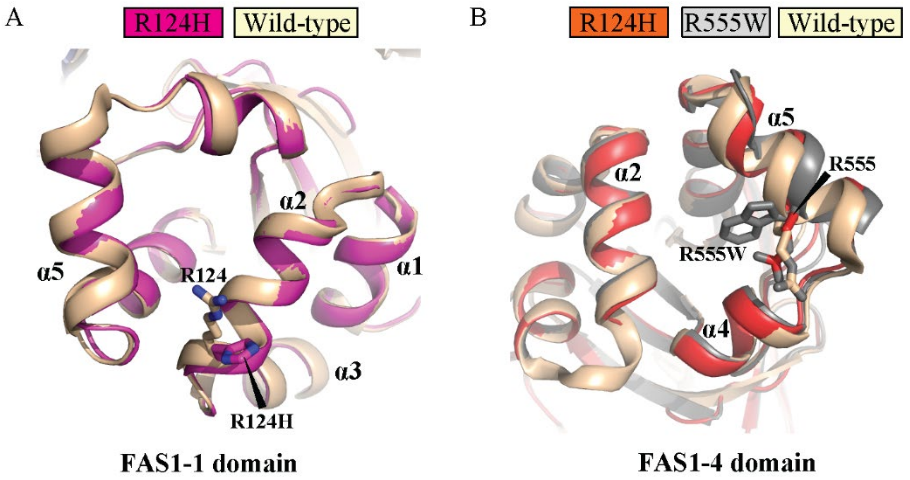

Figure S2. Mutation to R124H and R555W does not induce major structural changes. A) A comparison of their FAS1-1 domain reveals a virtual identical main chain conformation in R124H and wild-type TGFBIp. B) Comparison of FAS1-4 domains suggests minor changes in the two mutant proteins compared to the wild-type protein. This may well be due to the crystal packing interaction involving this domain from both the R124H and the R555W variants, which is absent in the crystal packing of wild-type TGFBIp.

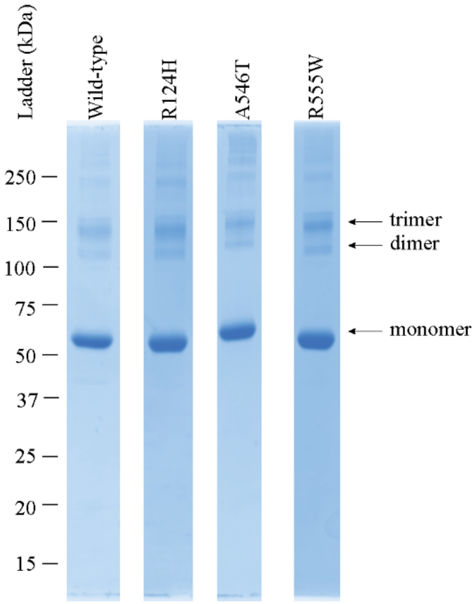

Figure S3. SDS-PAGE of DSSO cross-linked TGFBIp wild-type and mutants. Cross-linking of TGFBIp was carried out with 3.1 µM TGFBIp and 155 µM DSSO at room temperature for 30 min. The cross-linking reactions were quenched with 25 mM Tris-HCl pH 8.8 and subsequently analyzed by reducing SDS-PAGE. Bands containing monomers, dimers, and trimers of TGFBIp wild-type and mutants are indicated with arrows.

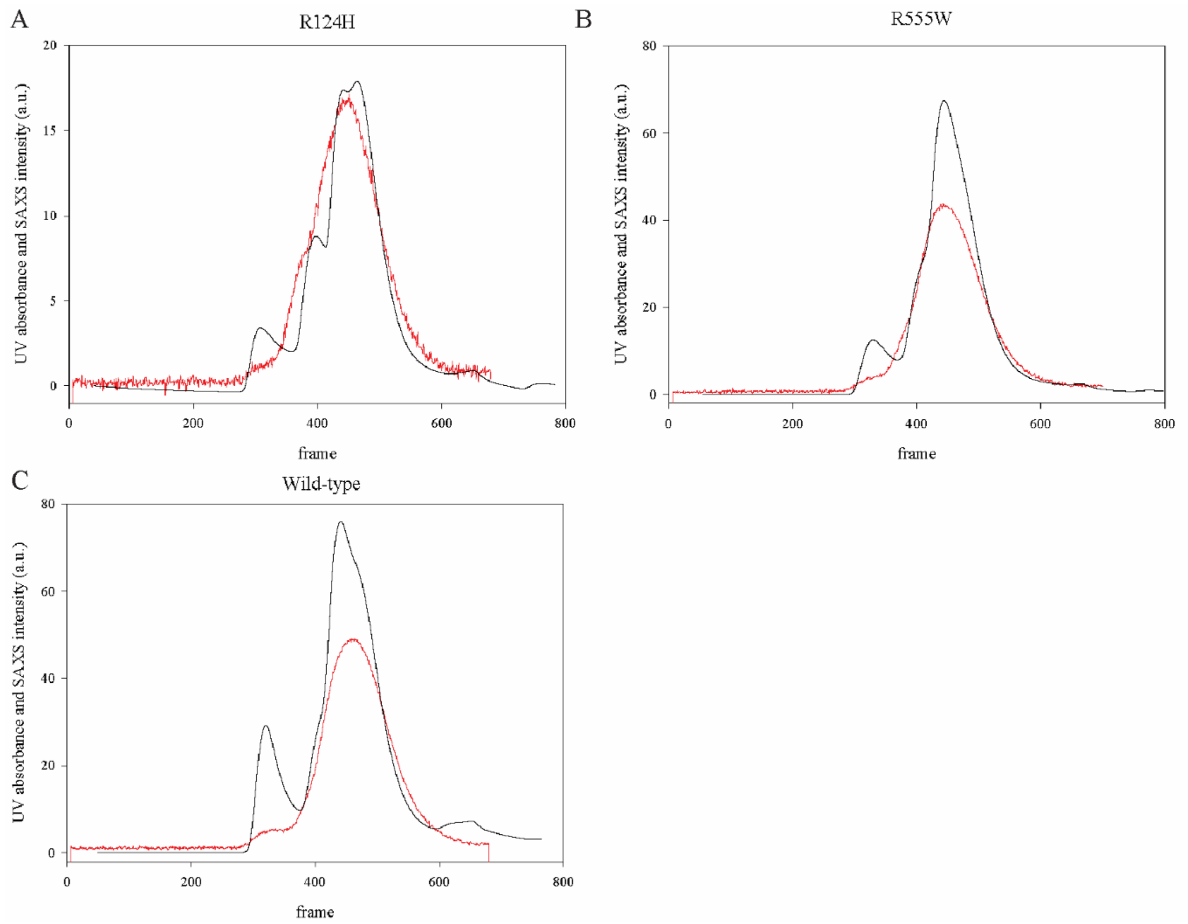

Figure S4. Elution profiles for R124H, R555W and wild-type from UV absorbance at 280 nm (black curve) and SAXS (red curve). The latter are obtained by adding the intensities for the lowest 100 q values.

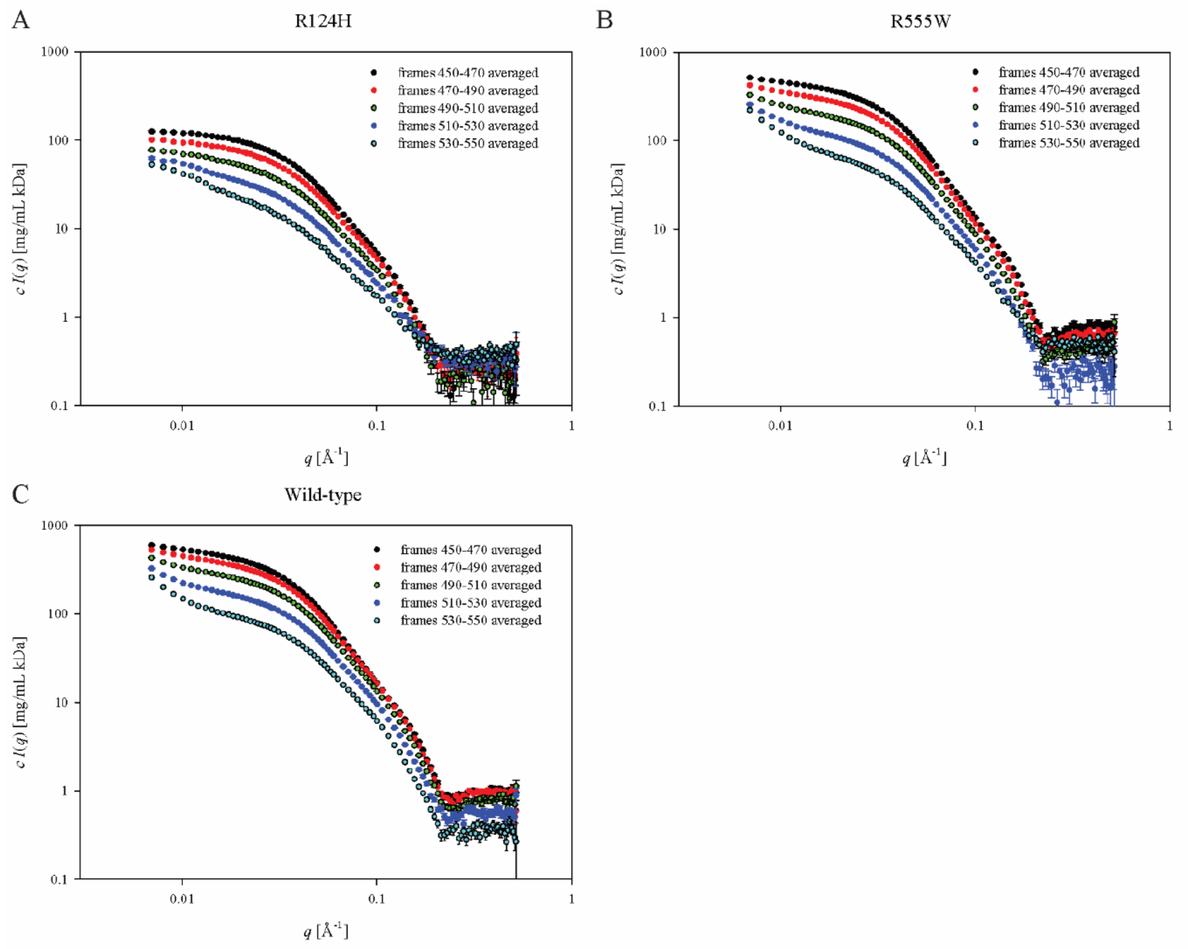

Figure S5. Buffer-subtracted SAXS data for the five fractions for each sample.

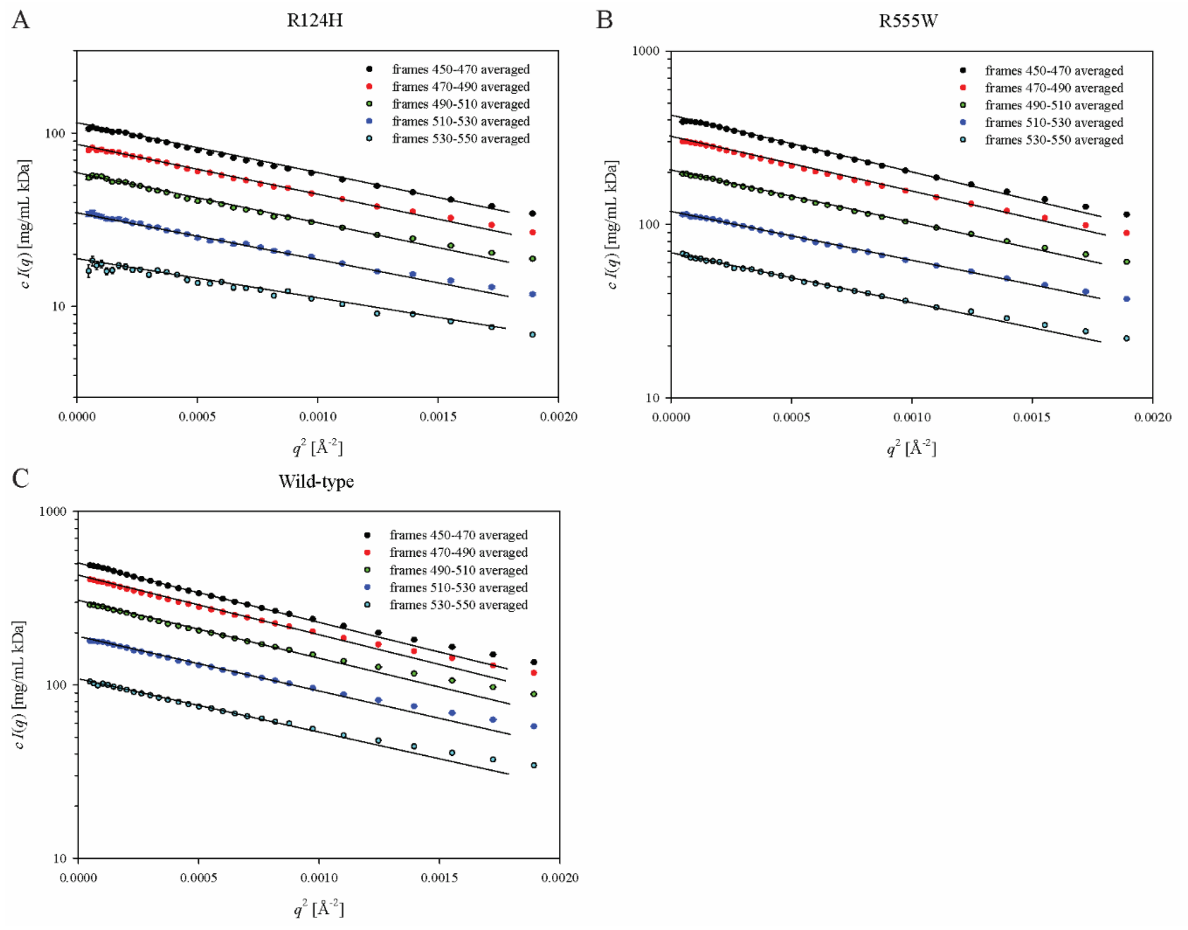

Figure S6. Guinier plots of SAXS data for the five fractions for each sample, where the scattering from the large species eluting late has been subtracted.

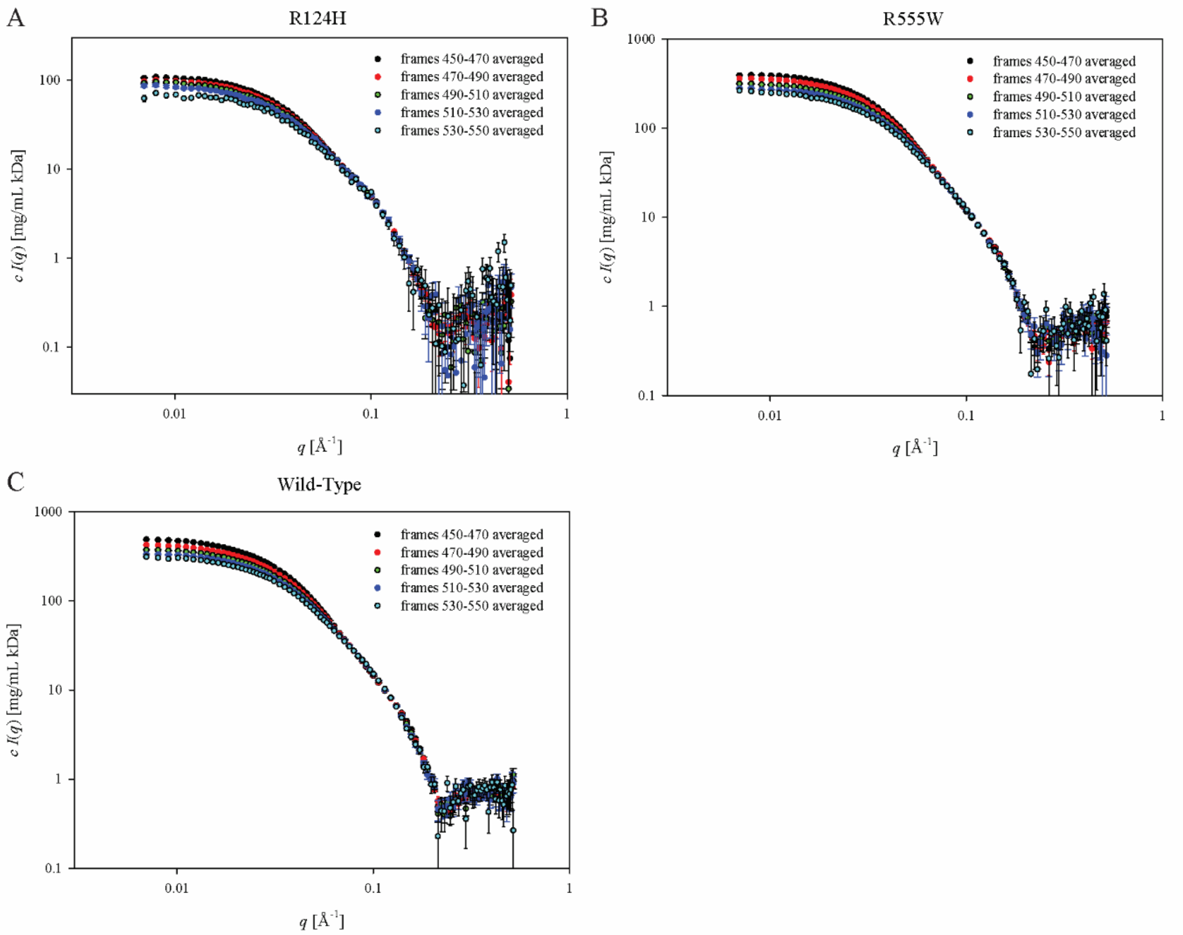

Figure S7. Plot of SAXS data for the five fractions for each sample, where the scattering from the large species eluting late has been subtracted and the data for each sample scaled to agree at high *q*.

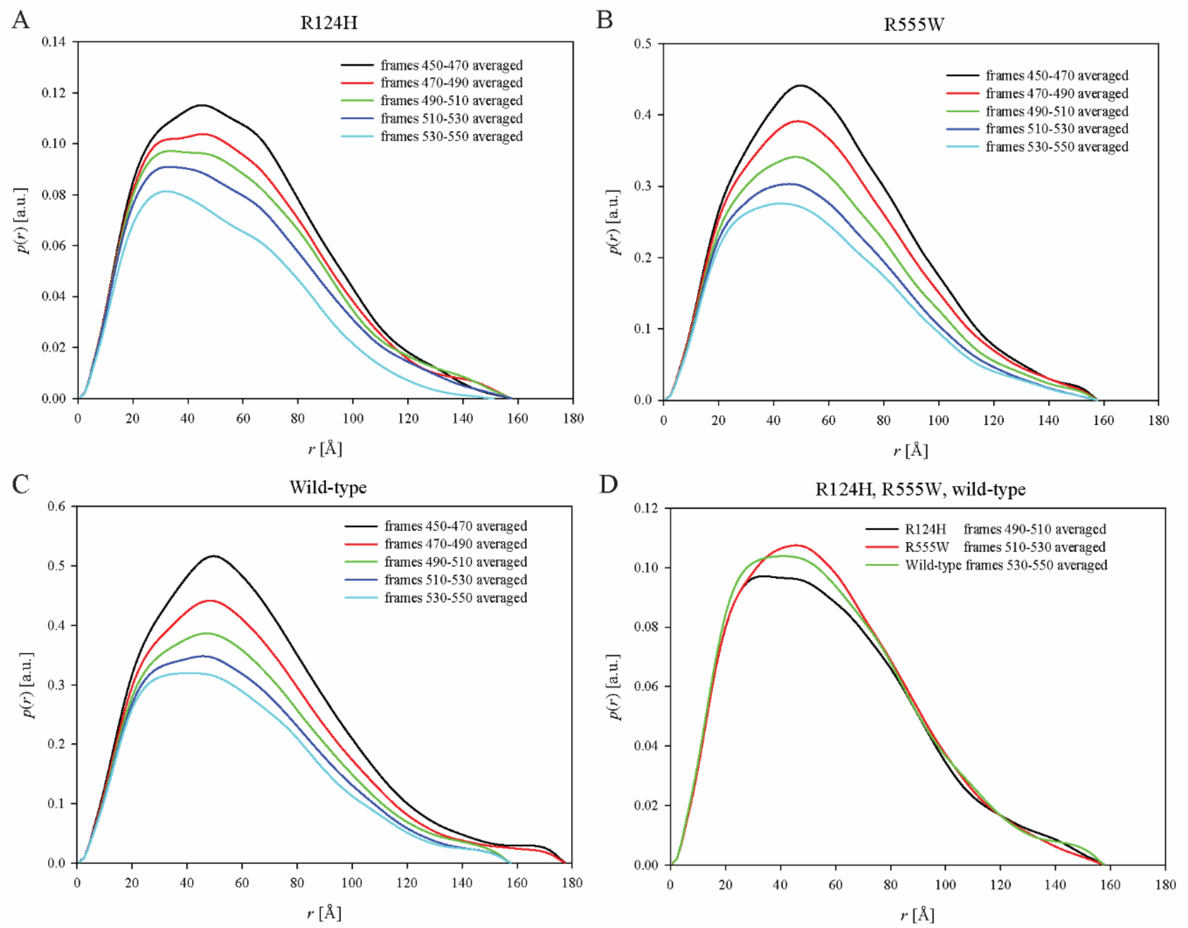

Figure S8. Distance distance distribution function $p(r)$ for the scaled data. A comparison of the functions for the R124H, R55W and wild-type for the fractions, no. 3 (frames 490-510), no. 4 (frames 510-530) and no. 5 (frames 530-550), respectively, are shown in the lower right corner.

Position A	Position B	Wild-type	R124H	A546T	R555W
60	72	x	x	x	x
60	90	x	x	x	x
60	219	x	x	x	x
66	90	x			
72	90	x	x	x	x
441	462	x	x	x	x
461	484				x
461	563			x	
461	590			x	
484	563			x	
563	570	x	x	x	x

Table S1. Intra-molecular cross-links.

Position A	Position B	Wild-type	R124H	A546T	R555W
72	596	x	x	x	x
90	563				x
90	596	x	x	x	x
127	596	x		x	x

Table S2. Inter-molecular cross-links.

Table S3. Values for forward scattering (arbitrary scale) and radius of gyration (Å) obtained from indirect Fourier transformation for the five different fractions for each sample.

R124H	frames averaged	*c I*(0) [*]			R_g [Å]		
	450-470	113.8	+/-	0.24	46.56	+/-	0.14
	470-490	104.2	+/-	0.32	46.56	+/-	0.20
	490-510	98.6	+/-	0.36	46.83	+/-	0.23
	510-530	89.4	+/-	0.53	45.70	+/-	0.39
	530-550	73.0	+/-	1.07	42.58	+/-	1.04
R555W							
	450-470	421.8	+/-	0.38	48.52	+/-	0.05
	470-490	379.4	+/-	0.39	48.16	+/-	0.06
	490-510	330.6	+/-	0.47	47.34	+/-	0.10
	510-530	293.0	+/-	0.77	46.26	+/-	0.17
	530-550	267.1	+/-	1.19	45.96	+/-	0.31
Wild-type	450-470	511.1	+/-	0.40	50.88	+/-	0.07
	470-490	440.8	+/-	0.47	50.01	+/-	0.08
	490-510	385.9	+/-	0.48	48.08	+/-	0.08
	510-530	348.4	+/-	0.62	47.19	+/-	0.13
	530-550	320.2	+/-	1.00	46.68	+/-	0.20

* *c I*(0): Forward intensity in mg/ml kDa for data scaled to the data set for frames 450-590 averaged.

Table S4: Distance values for restraints used during rigid-body refinement of the dimer models to the SAXS data (residues 72-596 and 90-596) and distances calculated for two other possible cross-links (residues 127-596 and 90-563) in Å for most representative models for the main clusters determined by the SAXS analysis. Average χ^2 values for the cluster are given and the models are indicated.

Cluster	72-595	90-596	127-596	90-563	$<\chi^2>$	Model		
R124H-1	14.0	26.4	44.5	41.2	3.6	N-in-arc	open	Coplanar
R124H-2	19.7	28.7	44.5	38.7	3.3	N-in-arc	closed	Coplanar
R124H-3	17.2	29.9	56.0	23.7	4.6	Arc-on-arc	opposite	Not quite coplanar
R555W-1	25.7	27.3	47.7	41.3	2.7	N-in-arc	closed	Coplanar
R555W-2	18.7	27.4	54.6	17.4	3.0	Arc-on-arc	opposite	Not quite coplanar
wild-type-1	14.2	31.2	41.3	48.6	4.0	C-in-arc	closed	Coplanar
wild-type-2	17.4	16.6	44.4	35.2	3.9	N-in-arc	closed	Not coplanar
wild-type-3	18.7	27.4	38.4	40.0	5.1	N-in-arc	open	Coplanar
